# Revisiting Metformin in the Management of Polycystic Ovary Syndrome (PCOS)

**DOI:** 10.7759/cureus.88216

**Published:** 2025-07-18

**Authors:** Venkata Madhavi Latha Telagarapu, Priyanka Devi Nallamothu, Venkata Nikhila Telagarapu, Srikar Raja Chowdary Nalluri, Saispoorthy Nathani, Chandu Priya Kopani, Vishnu Yarraguntla, Sriharika Vyasalapu

**Affiliations:** 1 Internal Medicine, Private Practice, New York, USA; 2 Public Health, Miller School of Medicine, University of Miami, Miami, USA; 3 Biomedical Engineering, University of Houston, Houston, USA; 4 Obstetrics and Gynecology, Sai Nikhila Clinic, Visakhapatnam, IND; 5 Surgery, Siddhartha Medical College, Vijayawada, IND; 6 Genomic Medicine, Cleveland State University/Cleveland Clinic, Cleveland, USA; 7 Medicine and Surgery, Kasturba Medical College, Manipal, IND

**Keywords:** anovulation, hyperandrogenism, hyperinsulinemia, hypothalamus, metformin, myo-inositol, obesity, oral contraceptive pills, pcos, sodium-glucose cotransporter-2 (sglt-2) inhibitors

## Abstract

Polycystic ovary syndrome (PCOS) is the most common endocrine disorder that significantly affects both obese and non-obese women of reproductive age. Besides genetic and epigenetic factors, obesity and insulin resistance (IR) play a key role in the pathogenesis of PCOS, contributing to its endocrine, reproductive, and metabolic manifestations. Even though metformin remains a well-established treatment in the management of PCOS, emerging therapies and integrative approaches warrant comparison. This narrative review aims to understand the pathophysiology of PCOS and compare the role of metformin with that of new emerging therapies, including sodium-glucose cotransporter-2 (SGLT-2) inhibitors, oral contraceptive pills (OCPs), inositol supplements, and lifestyle modifications, thereby empowering clinicians to provide personalized medicine based on each patient’s unique presentation. This review explores the safety, efficacy, and clinical outcomes of these interventions on weight reduction, hormonal balance, menstrual regularity, and metabolic outcomes in both obese and non-obese women with PCOS.

## Introduction and background

Obesity is widely recognized as a complex chronic condition characterized by excessive fat deposition in the body. According to the World Obesity Federation (WOF), one in eight people in the world was living with obesity, with over a billion people affected in 2024. The global prevalence of obesity in children and adolescents was 8.5%, with substantial variations across the countries, ranging from 0.4% in Vanuatu to 28.4% in Puerto Rico [1]. WOF predicts that the number of adults living with obesity will continue, with projections suggesting an increase from 0.81 billion in 2020 to 1.53 billion in 2035. The Centers for Disease Control and Prevention estimates that the prevalence of obesity among adults in the United States as of 2023 was 40.3%. Most recent available data report that the prevalence of obesity among children and adolescents aged 2-19 years in the United States increased from 16.9% in 2011-2012 to 19.7% from 2017-2020 [2]. The rise in childhood obesity is particularly concerning as it increases the likelihood of obesity persisting into adulthood [3].

The pathogenesis of primary obesity is a complex interplay of developmental, genetic, epigenetic, and environmental factors, including exposure to endocrine-disrupting chemicals. In addition, striking changes in lifestyle patterns over the past few decades, such as unhealthy dietary habits and reduced physical activity, socioeconomic status, ethnicity, psychosocial stress, and the gastrointestinal microbiome, have remarkably influenced the rise in obesity [4]. Obesity is associated with numerous short-term and long-term health conditions, including metabolic disorders such as type 2 diabetes mellitus, hypertension, and cardiovascular diseases [5]. The World Health Organization recognized obesity as a major public health problem due to its potential linkage to these chronic medical conditions, leading to significant morbidity, mortality, and economic burden on healthcare systems globally [6]. Studies have also demonstrated a significant association between obesity and various reproductive health problems, such as PCOS, infertility, and endometrial cancer [7,8].

PCOS is a complex, highly inherited, polygenic, and multifactorial endocrine disorder in women that can occur at any stage of reproductive life, with its onset commonly occurring during adolescence [9,10]. Almost 80% of women with PCOS are obese, and the prevalence rates are six- to sevenfold higher among those with morbid obesity [11]. Even though PCOS is typically considered a gynecological disorder characterized by androgen excess leading to hirsutism, anovulation, menstrual irregularities, infertility, and endometrial cancer, it also influences metabolic processes [10,12]. Due to its complex metabolic interactions, like obesity, insulin resistance (IR), increased risk of type 2 diabetes, and cardiovascular diseases, PCOS has been increasingly recognized as a multisystem illness. Hence, it is important to optimize the appropriate use of insulin-sensitizing drugs in the comprehensive management of PCOS.

This review explores the pathophysiology of IR in women with PCOS and aims to provide a comprehensive overview of insulin-sensitizing medications in managing the metabolic, endocrine, and reproductive effects of PCOS, with a particular focus on metformin. In addition, this narrative review also compares the efficacy of metformin with other evidence-based treatment modalities in both obese and non-obese PCOS phenotypes.

Pathophysiology of PCOS

**Figure 1:**
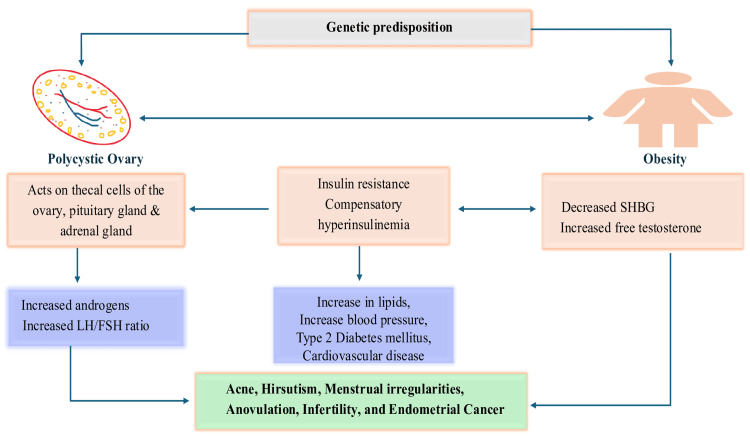
Pathophysiology of polycystic ovarian syndrome (PCOS): conceptual framework Schematic overview of the complex interrelationship between genetic predisposition, obesity, polycystic ovary, and their downstream metabolic and reproductive consequences. The illustration depicts how insulin resistance, hormonal imbalances, and metabolic changes contribute to clinical features associated with polycystic ovarian syndrome (PCOS). Image Credit: Figure created by the authors using original drawings and modified icons from Microsoft PowerPoint (Microsoft Corp., Redmond, WA, United States).

Given the central role of IR and obesity in the etiology of PCOS, understanding the underlying mechanisms of these conditions is essential for this review (Figure 1). 

In PCOS, persistent gonadotropin-releasing hormone (GnRH) secretion and an elevated luteinizing hormone (LH) to follicle-stimulating hormone (FSH) ratio contribute to hyperandrogenism and anovulation [12]. Excess androgens are closely linked to both IR and hyperinsulinemia (HI), which are recognized as key components in the pathophysiology of PCOS [10,12]. Elevated androgen levels promote the accumulation of abdominal adipose tissue and contribute to adipocyte insulin resistance in affected women [13]. Hyperandrogenism, in turn, fosters obesity, while IR and obesity further exacerbate hyperandrogenic symptoms, forming a self-reinforcing cycle that drives disease progression.

Although insulin receptor affinity remains comparable between women with PCOS and healthy controls, impaired insulin binding at the pancreatic β-cells and in adipose tissue reduces glucose uptake and insulin sensitivity in women with PCOS [12]. IR and HI are reported in approximately 65%-95% of women with PCOS, including around 75% of those who are lean [13]. Notably, imaging studies have shown that lean women with PCOS have a disproportionately higher amount of preperitoneal and visceral fat compared to weight-matched controls [14], suggesting that IR may be an intrinsic feature of PCOS, independent of body mass.

## Review

Search methodology

We followed a comprehensive and structured approach, utilizing electronic databases such as PubMed and Web of Science, to identify relevant literature for this narrative review. Key terms including “polycystic ovary syndrome,” “obesity,” and “treatment,” along with other related keywords, were combined using Boolean operators to optimize the search and focus on studies addressing the pathophysiology of PCOS, mechanism of action of drugs used in the management of PCOS and comparing the clinical implications of metformin, sodium-glucose cotransporter-2 (SGLT-2) inhibitors, oral contraceptive pills (OCPs), inositol, and lifestyle modifications (LSMs). Eligible studies included clinical trials, observational research, recent high-impact reviews, and meta-analyses published in English from 2007 to 2024. 

Insulin-sensitizing agents in PCOS

As IR and compensatory HI are the cornerstones in the pathophysiology of PCOS and have a potential impact on hyperandrogenism leading to ovulatory dysfunction, interventions targeting IR are considered a rational therapeutic approach. Insulin-sensitizing agents have emerged as a cutting-edge therapy for both obese and non-obese PCOS phenotypes, especially those with underlying IR. This narrative review highlights the latest clinical studies to guide treatment strategies for women with PCOS and IR, with an emphasis on metformin.

Cellular and Molecular Mechanisms of Metformin

Metformin is one of the widely used insulin-sensitizing agents belonging to the biguanide group and is considered the first-line therapy in the treatment of type 2 diabetes mellitus. In the 1990s, metformin was first shown to alleviate hyperandrogenism and has been used in the management of PCOS. Since then, metformin emerged as a potential drug in the management of PCOS due to its therapeutic efficacy among all age groups and phenotypes of PCOS, well-established safety profile, and relatively low cost. In this section, the mechanism of action of metformin in PCOS will be explained to broaden our understanding and underscore its clinical efficacy in improving metabolic, reproductive, and endocrine outcomes.

*Effects of Metformin on Insulin Sensitivity* 

Metformin enhances peripheral insulin sensitivity and lowers blood glucose levels primarily by inhibiting gluconeogenesis in the liver. It also increases the peripheral uptake of glucose and reduces intestinal glucose absorption. However, the risk of inducing hypoglycemia is minimal [15]. Studies conducted by LaMoia et al. [16] and Foretz et al. [17] updated the latest advances in the mechanisms of action of metformin. At the cellular level, metformin acts on hepatic mitochondria and inhibits mitochondrial respiratory chain complex I, leading to a reduction in intracellular adenosine triphosphate (ATP) synthesis and concomitant rise in cellular levels of adenosine monophosphate (AMP) [16]. Increased AMP levels decrease hepatic gluconeogenesis. Metformin-induced increase in the AMP-ATP ratio activates AMP-activated protein kinase (AMPK), a key regulator of cellular energy homeostasis [17]. Activation of AMPK promotes the translocation of glucose transporter (GLUT) proteins toward the cell membrane and facilitates glucose uptake [18]. In addition, mitochondrial respiratory chain complex I inhibited by metformin modulates cell-specific inflammatory processes by both AMPK-independent and AMPK-dependent mechanisms [17]. Moreover, AMPK activated by metformin stimulates mitochondrial fat oxidation and inhibits lipogenesis in the liver, decreasing hepatic lipid accumulation. This reduction in lipotoxicity further contributes to improved insulin sensitivity [16,19]. 

Effect of Metformin on Excess Androgen Levels

Increased expression of 17α-hydroxylase/17,20 lyase (CYP17A1), cytochrome P450c17α, and 3β-hydroxysteroid dehydrogenase type 2 (HSD3B2) in the polycystic ovarian cells has been implicated in the pathogenesis of hyperandrogenism in women with PCOS [20,21]. Cytochrome P450c17α possesses both 17 alpha-hydroxylase and 17,20-lyase activities and mediates the production of 17 alpha-hydroxyprogesterone, a key intermediate in androgen biosynthesis [21]. Metformin inhibits androgen production by modulating the activity of HSD3B2 and CYP17A1 mechanisms, along with mitochondrial complex 1 inhibition, independent of AMPK signaling [20]. In an experimental study, Nestler and Jakubowicz [21] concluded that metformin reduces cytochrome P450c17α in the ovaries and alleviates hyperandrogenism.

A systematic review and meta-analysis conducted by Barba et al. examined 20 studies and found a statistically significant increase in the circulating levels of androgens and sex hormone-binding globulin (SHBG) in PCOS women treated with metformin. However, inference regarding the effects of metformin on the levels of androgens and SHBG in healthy women is inconclusive [22].

Effect of Metformin on Menstrual Regulation and Ovulation

A study conducted by Jonard and Dewailly revealed that intra‐ovarian hyperandrogenism may promote early follicular growth [23]. However, hyperinsulinism and insulin resistance amplify intra‐ovarian hyperandrogenism and induce excessive early follicular growth, ultimately resulting in follicular arrest in women with PCOS [23]. Another prospective comparative experimental study by Stanek et al. also reported that insulin and insulin-like growth factors (IGF-I and IGF-II), together with LH, stimulate vascular endothelial growth factor production in granulosa cells, which are particularly sensitive to insulin, leading to abnormal follicular angiogenesis [24]. 

In addition to its insulin-lowering effect, metformin decreases serum-free testosterone and LH-driven ovarian androgen synthesis, thereby fostering a less androgenic intraovarian environment [21]. This hormonal shift enhances follicular responsiveness to gonadotropins. Metformin further levels the bioavailability and biological activity of insulin-mediated IGF-I, thereby mitigating its pro-androgenic effects on theca cells. Additionally, it increases the levels of IGF-binding proteins (IGFBPs), which bind free IGF-I. This modulation of the IGF axis reduces premature luteinization, enhances oocyte maturity, and promotes follicular development [25]. Metformin helps reduce the levels of VEGF and platelet-derived growth factor (PDGF) and improves ovarian morphology by decreasing cystic structures and increasing the number of antral follicles and corpora lutea, ultimately promoting the restoration of the ovulatory cycles [25].

Effect of Metformin on Weight Management

Beyond its glucose-lowering effects, metformin facilitates modest weight reduction through a multi-tissue mechanism involving central appetite regulation, modulation of gut hormones, and metabolic reprogramming of adipose and hepatic tissues. Given the central role of obesity in the pathophysiology of PCOS, weight loss represents a critical therapeutic goal. Metformin-induced weight loss is largely attributed to its actions on hypothalamic appetite centers within the central nervous system (CNS) and alterations in the gut microbiome [15]. Malin et al. [26] and Yerevanian and Soukas [15] have explored how metformin suppresses appetite and limits fat accumulation in peripheral tissues. At the CNS level, metformin inhibits hypothalamic AMPK activity, thereby reducing the expression of orexigenic neuropeptides such as neuropeptide Y (NPY) and agouti-related peptide (AgRP), while enhancing anorexigenic pathways mediated by pro-opiomelanocortin (POMC) [15,26]. These neuroendocrine changes improve leptin and insulin sensitivity, increase incretin production, and enhance vagal signaling, collectively contributing to enhanced satiety and decreased food intake.

Additionally, LaMoia and Shulman demonstrated that metformin reduces visceral and ectopic fat accumulation in hepatic and skeletal muscle tissues by activating AMPK-dependent pathways that promote fatty acid oxidation and inhibit lipogenesis [15,16,26].

Yerevanian and Soukas also highlighted the impact of metformin on appetite regulation via the gut and gut-brain axis [15]. Metformin modulates bile acid absorption through interactions with farnesoid X receptors (FXR) and influences the secretion of anorexigenic peptides, such as glucagon-like peptide-1 (GLP-1) and peptide YY (PYY) [15].

In a 2023 systematic review of 13 clinical trials and observational studies, Pavlo et al. [27] investigated the impact of metformin on human gut microbiota composition and diversity. The review identified significant alterations in several bacterial genera across various phyla, although findings on overall diversity were inconsistent. For instance, *Akkermansia *abundance increased in two studies but decreased in two others; *Escherichia* levels increased in two studies, and *Bifidobacteria* (Actinobacteria phylum) were elevated in another. A decline in *Adlercreutzia *was noted in one study. While an increase in *Escherichia coli* has been associated with inflammation and insulin resistance, *Akkermansia muciniphila *appears to support glucose homeostasis and gut barrier integrity [27]. Based on these findings, Pavlo et al. concluded that metformin-induced changes in gut microbiota may play a role in the development and management of insulin resistance [27].

Therapeutic Implications of Metformin Use in Obese and Non-obese PCOS Phenotypes

Metformin demonstrates differential efficacy in the management of PCOS depending on body mass index (BMI), with distinct therapeutic outcomes in obese and non-obese women. These differences are particularly evident in metabolic improvements and reproductive function, including ovulatory and menstrual regulation.

In a randomized, double-blinded, placebo-controlled crossover trial, compared to placebo, metformin has been shown to significantly reduce body weight, fasting plasma glucose, insulin levels, testosterone, and the homeostatic model assessment of insulin resistance (HOMA) index in obese women with PCOS and BMI ≥ 30. Women with a BMI < 30 also experienced a reduction in insulin levels. However, the overall metabolic changes were less pronounced [28].

Sharpe et al. updated a previous review by including 41 randomized controlled trials encompassing 4,552 women, examining the efficacy of metformin alone or in combination with clomiphene citrate (CC), letrozole, and laparoscopic ovarian drilling (LOD) in improving reproductive outcomes in women with PCOS [29]. The findings highlighted a significant influence of BMI on treatment outcomes. Among obese women treated with metformin, lower rates of clinical pregnancy (OR 0.34; 95% CI: 0.21-0.55; I² = 0%; two studies) and ovulation (OR 0.29; 95% CI: 0.20-0.43; I² = 0%; two studies) were observed. In contrast, non-obese women demonstrated higher pregnancy rates (OR 1.56; 95% CI: 1.06-2.29; I² = 26%; six studies), although there was no consistent difference in ovulation rates. However, the authors noted that the overall quality of evidence was low. A separate study corroborated these findings, reporting that non-obese women experienced higher ovulation rates and greater improvements in menstrual regularity following metformin therapy, while obese women were 77.9% less likely to ovulate under the same treatment conditions [30].

In a prospective clinical interventional study evaluating metformin’s impact on lipid profiles in obese and non-obese women with PCOS, both groups exhibited increased high-density lipoprotein (HDL) levels; however, the increase was statistically significant only among non-obese women (p < 0.001) [31]. While total cholesterol levels significantly decreased in the obese cohort, no significant change was seen among non-obese participants. Low-density lipoprotein (LDL) and triglyceride (TAG) levels remained unchanged after six months of metformin therapy in both groups.

Similarly, a study conducted at the Government Medical College, Patiala, reported a significant reduction in lipid parameters following six months of metformin treatment in women with PCOS. The study found a 9.8% decrease in serum cholesterol, a 16.19% reduction in mean LDL, and a notable decline in triglycerides. Additionally, HDL levels increased significantly by 12.08% from baseline [32].

Efficacy of LSM alone versus in combination with metformin in PCOS

Due to its positive impact on weight management and insulin resistance and favorable safety profile, LSM, including healthy dietary changes and increased physical activity, is widely recommended as the first-line treatment for PCOS. However, adherence to LSM is often suboptimal, highlighting the potential benefit of combining LSM with pharmacologic interventions such as metformin. This approach may be particularly advantageous for women presenting with more severe obesity or marked insulin resistance. Several studies have investigated the comparative effectiveness of LSM alone, metformin monotherapy, and their combination across various metabolic and reproductive parameters in women with PCOS.

A systematic review and meta-analysis by Naderpoor et al. analyzed 12 randomized controlled trials involving 608 women with PCOS, assessing the effects of LSM and metformin (with or without placebo) [33]. The findings demonstrated that the combination of LSM and metformin resulted in significantly greater reductions in BMI and subcutaneous adipose tissue, along with improved menstrual regularity, over six months compared to LSM with placebo. Although weight and BMI were similar between groups after six months, testosterone levels were lower in participants receiving metformin alone compared to those on LSM with or without placebo. No consistent differences were observed in clinical manifestations such as acne or hirsutism across the intervention arms [33].

Additionally, the study found that women in the LSM plus metformin group experienced a higher number of menstrual cycles over six months compared to those receiving LSM with or without placebo. However, no statistically significant differences in menstrual improvement were identified when comparing LSM alone to metformin alone, or LSM alone to the combination of LSM and metformin [34].

Metformin versus other medications

Metformin Versus SGLT-2 Inhibitors in the Management of PCOS

Emerging research, including preclinical and early-phase clinical studies, has shown the potential benefits of new medications like glucagon-like peptide 1 (GLP1) agonists and SGLT-2 inhibitors for PCOS [35]. As the early studies are showing encouraging results, we are comparing the role of metformin with the limited available data on GLP agonists and SGLT-2 inhibitors in the management of PCOS.

SGLT-2 inhibitors, a newer class of oral hypoglycemic drugs, reduce glucose reabsorption in the proximal convoluted tubule and lower blood glucose levels by promoting renal excretion of glucose. Beyond their antihyperglycemic effects, SGLT-2 inhibitors exhibit potential cardiovascular and metabolic benefits, rendering them compelling options for the therapeutic management of PCOS [35]. The review study by Porth et al. concluded that SGLT-2 inhibitors have been shown to improve glycemic indices, reduce body weight and total fat mass, lower total testosterone and dehydroepiandrosterone sulfate (DHEAS) levels, and restore menstrual cycles in patients with PCOS [35]. SGLT-2 inhibitors have a similar effect on improving menstrual cycles as metformin when compared to a placebo or standard of care in patients with PCOS. However, unlike metformin, their use is associated with a higher risk of urinary tract infections and ketoacidosis. SGLT-2 inhibitors may be better tolerated in certain patient populations, making them a potentially suitable option for selected adolescents based on individual risk profiles.

A prospective, randomized, open-label (ratio 1:1) noninferiority trial conducted at the Department of Endocrinology, Shanghai, compared the safety and efficacy of canagliflozin, SGLT-2 inhibitors, to metformin in PCOS women with IR treated for 12 weeks. Both canagliflozin and metformin significantly improved menstrual patterns, reduced body weight and total fat mass, and decreased triglyceride levels. The results of the study reported significant improvement in menstrual patterns, a decrease in body weight and total fat mass, and reduced triglyceride levels among both canagliflozin and metformin groups [36]. In addition, the study identified a significant advantage with canagliflozin in the reduction of serum uric acid and dehydroepiandrosterone sulfate levels when compared with the metformin group.

However, a randomized open-label study was conducted in women with PCOS treated with empagliflozin, another SGLT-2 inhibitor, and compared to metformin. The study showed significant improvement in anthropometric parameters and body composition, including weight, BMI, waist, and hip circumferences in obese and overweight women with PCOS after 12 weeks of treatment with empagliflozin compared to metformin [37]. However, no significant changes were observed in hormonal or metabolic parameters.

Metformin Versus OCPs in the Management of PCOS

OCPs are considered the mainstay of pharmacologic therapy for women with PCOS to mitigate hyperandrogenic symptoms such as acne and hirsutism, regularize the menstrual cycles, and protect the endometrium from hyperplasia secondary to unopposed estrogen action.

To study the effect of the combined oral contraceptives (COCs) with or without metformin in the management of polycystic ovary syndrome, Teede et al. conducted a systematic review with meta-analysis including 56 studies [38]. The meta-analysis demonstrated that metformin was better than OCPs for improving BMI and fasting insulin, and OCPs were found to be better than metformin for menstrual regulation. However, metformin alone has been proven to be effective for adult women, especially those with a BMI ≥ 25 kg/m^2^ in weight management, hormonal, and metabolic outcomes [38]. A Cochrane review updated in 2020 observed that metformin may be less effective in improving hirsutism compared to the OCP in PCOS women with a BMI of 25-30 kg/m^2^ and uncertain among women with a BMI > 30 kg/m^2^ when compared to those with a BMI < 25 kg/m^2^. Moreover, the study revealed that metformin may increase severe gastrointestinal adverse events compared to the OCPS and decrease the severe adverse events stopping medication [39].

A randomized controlled trial evaluated the effects of metformin and COCs in adolescent PCOS women through a 24-month follow-up period [40]. The study reported that metformin and COC have comparable therapeutic effectiveness on menstrual cycle regularity and hirsutism. In addition, the study also found a significant loss of weight in the metformin group, while a non-significant gain in weight was noted in the COC group. While OCPs can effectively regulate menstrual cycles and improve hirsutism and acne in PCOS patients, their potential for weight gain, worsening insulin resistance, and low-grade inflammation causing atherosclerotic changes make it important to do a risk-benefit assessment before initiating long-term therapy.

Metformin Versus Myo-inositol (MYO) in the Management of PCOS

MYO has emerged as a promising alternative to metformin in the management of PCOS, particularly due to its favorable safety profile. While MYO has demonstrated the ability to reduce BMI, its impact is less consistent when compared to metformin, particularly in women with higher baseline BMI. Some studies report comparable reductions in weight between MYO and metformin. Greff et al. assessed the safety and efficacy of inositol in PCOS by conducting a systematic review and meta-analysis of 26 randomized controlled trials, including data from 1691 patients treated with inositol, metformin, and placebo [41]. The study showed that inositol induced a greater decrease in BMI, free testosterone, total testosterone, androstenedione, glucose, and AUC insulin compared to placebo [41]. Moreover, MYO, when compared to metformin, also demonstrated a comparable efficacy regarding cycle normalization and total testosterone levels, with a significant increase in SHBG levels in the study. Another randomized controlled trial found that compared with metformin after the 12-week intervention, MYO significantly decreased serum total testosterone, modified Ferriman-Gallwey (mF-G) scores for hirsutism, and serum high-sensitivity C-reactive protein (hs-CRP) levels and gene expression of IL-1 [42].

The safety and efficacy of metformin in comparison with other treatment modalities are summarized in Table 1.

**Table 1:** Comparative evaluation of safety and efficacy of different therapeutic modalities in PCOS management

Outcome	Metformin alone	LSM alone	LSM + metformin	Oral contraceptives	SGLT-2 inhibitors	Myo-inositol
BMI reduction	Moderate	Moderate	Greater reduction	Non-significant weight gain	Significantly greater	Less consistent results
Subcutaneous fat reduction	Mild	Mild	Significantly greater	Less consistent results	Preferential fat loss while preserving lean mass better	Mild
Testosterone level	Lower than LSM	No significant change	Similar to metformin	Significantly greater	Similar to metformin	Similar to metformin
Menstrual irregularity	Improved	Improved	Improved more than LSM alone	Improved better than metformin	Similar to metformin	Similar to metformin
Ovulation rate	Improved	Improved	Possibly improved (not significant)	Anovulation	Improved	Similar to metformin
Acne/hirsutism	Improved	Improved	Improved	Greater improvement	Greater improvement	Similar to metformin
Compliance	Moderate	Often low	May enhance adherence	Similar dropouts	More than 90%	Similar to metformin
Complications	GI distress, hypoglycemia	None	Hypoglycemia, GI distress, B12 deficiency	Deterioration of metabolic syndrome/worsening of insulin resistance	UTI, ketoacidosis	No long-term effects

Clinical implications

Adhering to metformin therapy can be particularly challenging for adolescents due to a lack of universally standardized metformin dosage or an optimal treatment duration protocol. Long-term controlled studies are needed to establish definitive safety, efficacy, and dosing strategies among this population. Metformin is commonly associated with gastrointestinal intolerance, causing nausea, flatulence, diarrhea, indigestion, vomiting, and abdominal discomfort, with diarrhea and nausea being the most common [43]. Several observational studies and meta-analyses have reported a significant association between chronic metformin treatment and an increased prevalence of malabsorption and deficiency of vitamin B12. Periodical monitoring and supplementation of vitamin B12 are recommended during chronic therapy to prevent megaloblastic anemia, progressive axonal demyelination, and peripheral neuropathy [44]. As metformin remains a cornerstone in the management of reproductive health in PCOS, further high-quality studies are warranted to explore its impact on pregnancy outcomes, including miscarriage rates, teratogenicity, and live birth rates.

Long-term use of OCPs can cause challenging side effects, including weight gain, metabolic issues, withdrawal bleeding with missed doses, decreased bone mineral density, and cardiovascular effects. Because of these side effects, OCPs are not the first choice for adolescents. Considering its better safety profile, metformin may help those primarily concerned with hirsutism and acne, while OCPs should be reserved for those with menstrual cycle irregularities. Evidence shows that SGLT-2 inhibitors can significantly reduce BMI, acne, and hirsutism, with similar results in controlling testosterone levels, regulating menstrual cycles, and enhancing ovulation. However, clinical data supporting SGLT-2 inhibitors are limited, and they have been associated with UTI and ketoacidosis. Larger studies are needed to confirm their long-term safety. MYO has demonstrated comparable efficacy to metformin, with no long-term adverse effects reported in current studies.

## Conclusions

PCOS is a prevalent endocrinopathy with multisystem involvement, predominantly affecting women of reproductive age. Given the central roles of IR and obesity in its pathophysiology, addressing these metabolic abnormalities is essential in optimizing treatment strategies. This review supports the continued use of metformin as a safe, effective, and cost-efficient therapeutic option for both obese and non-obese women with PCOS, particularly for improving metabolic profiles and reproductive outcomes with limited side effects. Evidence suggests that metformin’s benefits are further enhanced when used in conjunction with LSMs, including dietary changes, physical activity, and weight management. This combination approach may improve clinical outcomes while allowing for lower pharmacologic dosing. MYO is showing results comparable to metformin with no side effects. Large and long-term trials are necessary to further enhance the safety of emerging therapies.

Clinicians should weigh the risks and benefits of available treatment options in the management of PCOS. Considering the patient’s age, BMI, presenting symptoms, personal and family risk factors, and the patient's choice, pharmacological therapy should be tailored for every patient. LSMs, including healthy dietary habits and regular physical activity, are strongly recommended along with medication for women with PCOS, as they play a key role in improving insulin resistance.
